# Immunogenicity and Cross-Reactivity of Rhesus Adenoviral Vectors

**DOI:** 10.1128/JVI.00159-18

**Published:** 2018-05-14

**Authors:** M. Justin Iampietro, Rafael A. Larocca, Nicholas M. Provine, Peter Abbink, Zi Han Kang, Christine A. Bricault, Dan H. Barouch

**Affiliations:** a Center for Virology and Vaccine Research, Beth Israel Deaconess Medical Center, Harvard Medical School, Boston, Massachusetts, USA; b Ragon Institute of MGH, MIT and Harvard, Boston, Massachusetts, USA; Emory University

**Keywords:** HIV, adenovirus, rhesus, simian immunodeficiency virus, vector

## Abstract

Adenovirus (Ad) vectors are being investigated as vaccine candidates, but baseline antivector immunity exists in human populations to both human Ad (HuAd) and chimpanzee Ad (ChAd) vectors. In this study, we investigated the immunogenicity and cross-reactivity of a panel of recently described rhesus adenoviral (RhAd) vectors. RhAd vectors elicited T cells with low exhaustion markers and robust anamnestic potential. Moreover, RhAd vector immunogenicity was unaffected by high levels of preexisting anti-HuAd immunity. Both HuAd/RhAd and RhAd/RhAd prime-boost vaccine regimens were highly immunogenic, despite a degree of cross-reactive neutralizing antibodies (NAbs) between phylogenetically related RhAd vectors. We observed extensive vector-specific cross-reactive CD4 T cell responses and more limited CD8 T cell responses between RhAd and HuAd vectors, but the impact of vector-specific cellular responses was far less than that of vector-specific NAbs. These data suggest the potential utility of RhAd vectors and define novel heterologous prime-boost strategies for vaccine development.

**IMPORTANCE** To date, most adenoviral vectors developed for vaccination have been HuAds from species B, C, D, and E, and human populations display moderate to high levels of preexisting immunity. There is a clinical need for new adenoviral vectors that are not hindered by preexisting immunity. Moreover, the development of RhAd vector vaccines expands our ability to vaccinate against multiple pathogens in a population that may have received other HuAd or ChAd vectors. We evaluated the immunogenicity and cross-reactivity of RhAd vectors, which belong to the poorly described adenovirus species G. These vectors induced robust cellular and humoral immune responses and were not hampered by preexisting anti-HuAd vector immunity. Such properties make RhAd vectors attractive as potential vaccine vectors.

## INTRODUCTION

Adenoviruses (Ads) have been explored as vaccine vectors due to their safety profile and immunogenicity (1, 2). However, baseline neutralizing antibodies (NAbs) exist in human populations to commonly used human Ad (HuAd) and many chimpanzee Ad (ChAd) vectors (1, 3). High titers of vector-specific NAbs have been shown to attenuate the humoral and cellular immune responses elicited by these vectors due to neutralization of the vaccine vector following immunization (2, 4–6). Developing Ad vectors for which there is minimal to no baseline seroprevalence is therefore important. Vector-specific humoral immunity is largely serotype specific, whereas vector-specific cellular immunity has been reported to be highly cross-reactive among serotypes (1–4, 7–16). These considerations have led to interest in rare human and nonhuman adenoviral vectors in an effort to circumvent baseline immunity (7, 16, 17).

Many different adenovirus serotypes exist, and most human adenoviruses are from species A to F (18). ChAds typically fall into species B, C, or E and thus are phylogenetically similar to human Ads (19). Our laboratory and others have shown that the Ad serotype impacts the phenotype of the resulting innate and adaptive immune responses induced by these vectors (20–22). These observations highlight the importance of understanding and characterizing each Ad vector serotype for use as potential vaccine vectors.

We previously described three rhesus adenoviruses (RhAds) that cluster into the poorly characterized adenovirus species G (18). In addition, we recently reported 14 additional RhAd vectors that also cluster with species G (23). In the present study, we investigated the immunogenicity and vector-specific cross-reactivity of a panel of RhAd vectors. We show that RhAd vectors potently induce both humoral and cellular immune responses and that RhAd vectors are unaffected by high levels of preexisting HuAd-specific immunity. We also assessed the extent of humoral and cellular cross-reactivity between RhAd and HuAd vectors and between different RhAd vectors. Our data suggest the potential of RhAd vectors for vaccine development and outline strategies for the development of robust heterologous prime-boost regimens.

## RESULTS

### Cellular immune phenotypes induced by rhesus adenoviruses.

**FIG 1:**
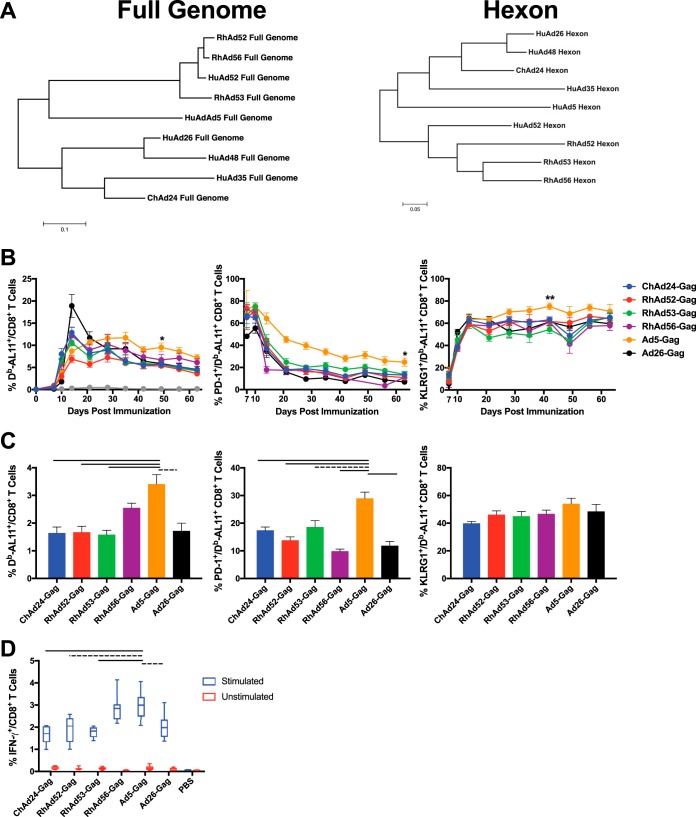
RhAd vector-induced cellular immunologic phenotype. Mice were immunized i.m. with 10^9^ vp of the indicated adenoviral vectors. (A) Phylogenetic trees showing the full-genome (left) and hexon (right) relationships among various HuAds, ChAd24, RhAd52, RhAd53, and RhAd56. (B) Longitudinal analysis of D^b^/AL11 tetramer-positive, PD-1^+^, and KLRG1^+^ CD8^+^ T cells from PBMCs. (C) Frequency of D^b^/AL11^+^, PD-1^+^, and KLRG1^+^ CD8^+^ T cells from splenocytes. (D) Frequency of IFN-γ^+^ CD8^+^ T cells from splenocytes. Blue bars indicate splenocytes stimulated with the SIV_mac_239 Gag peptide pool, and red bars are unstimulated samples. Box-and-whisker plots indicate minimum and maximum values. For all experiments, data are for 8 to 12 mice per group. Lines above the graphs denote significance: solid bars, *P* < 0.0001; dotted lines, *P* < 0.01. Error bars indicate the standard error of the mean (SEM).

We initiated studies to investigate the immunogenicity of a panel of RhAd vectors in comparison with that of the chimpanzee Ad serotype 24 (ChAd24) and human Ad5 and Ad26 vectors (Fig. 1A) (18). Groups of C57BL/6 mice (*n* = 8 to 12/group) were injected intramuscularly (i.m.) with 10^9^ viral particles (vp) of ChAd24-Gag, RhAd52-Gag, RhAd53-Gag, RhAd56-Gag, Ad5-Gag, or Ad26-Gag, and cellular immune responses were assessed by D^b^/AL11 tetramer binding and intracellular staining (ICS) assays (24).

As shown in Fig. 1B, all vectors were immunogenic, although Ad5-Gag induced the highest frequency of D^b^/AL11^+^ CD8^+^ T cells at the set point after day 28, with a mean of 9.5% tetramer-positive CD8^+^ T cells for Ad5-Gag at day 49 compared to a mean of 5.3% to 6.7% for all other vectors (Ad5-Gag versus Ad26-Gag, RhAd52-Gag, RhAd53-Gag, and ChAd24-Gag, *P* < 0.01). However, Ad26-Gag, ChAd24-Gag, and all RhAd-Gag vectors expressed lower levels of PD-1 (3.6 to 17.1%) than did Ad5-Gag (25.9%) at the terminal time point (Ad5-Gag versus all other vectors, *P* < 0.03) (22, 25). Moreover, Ad5-Gag vaccination resulted in higher expression of KLRG1-positive (KLRG1^+^) vaccine-elicited T cells than Ad26-Gag, ChAd24-Gag, and RhAd-Gag vector vaccination (Ad5-Gag versus all other vectors on day 42, *P* < 0.01), which suggests a more effector-like, rather than memory-like, phenotype (26, 27). The responses in the spleen were similar to those in peripheral blood mononuclear cells (PBMCs) on day 63 (Fig. 1C). ICS on splenocytes also showed that Ad5-Gag and RhAd56-Gag induced the highest frequencies of gamma interferon (IFN-γ)-positive (IFN-γ^+^) CD8^+^ T cells (Fig. 1D). These data suggest that the RhAd vectors induce T cells with a phenotype similar to that of T cells induced by Ad26 and different than the high-frequency, exhausted, effector phenotype of T cells induced by Ad5 (22, 25).

### Cellular immunogenicity of RhAd vectors in prime-boost regimens.

**FIG 2:**
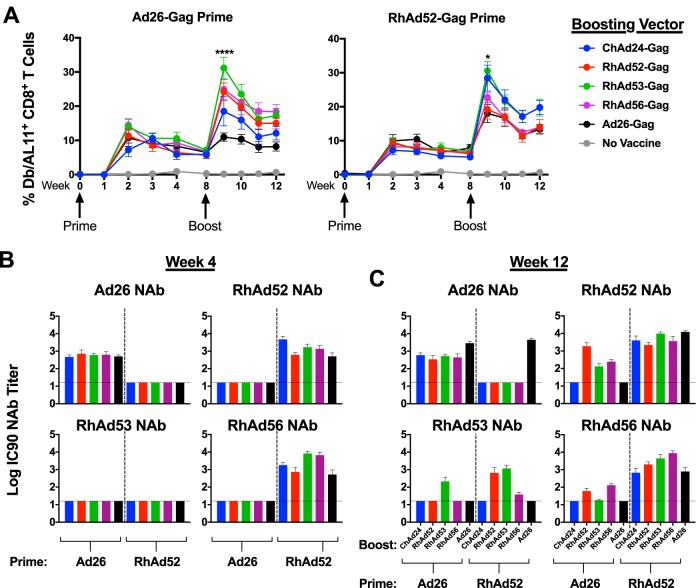
RhAd vectors in prime-boost regimens. C57BL/6 mice (*n* = 8 to 10/group) were primed with 10^9^ vp of the indicated adenoviral vector. After 8 weeks, mice were boosted with the vector shown in the key. (A) Longitudinal analysis of D^b^/AL11^+^ CD8^+^ T cells in PBMCs of vaccinated mice. For Ad26 prime, *P* was <0.0001 RhAd53 versus Ad26 (****) and *P* was equal to 0.0464 for RhAd53 versus ChAd24 (*); for RhAd52 prime, *P* was equal to 0.0142 for RhAd53 versus Ad26 (*). (B) Ad-specific neutralization titers 4 weeks after prime, before boosting vaccinations were administered. (C) Ad-specific neutralization titers 4 weeks after the boosting vaccinations were administered. Dotted lines indicate the limit of detection. Error bars indicate the standard error of the mean (SEM). For tetramer analysis, 8 to 10 mice per group were used, and for neutralization data, 4 to 8 mice per group were used. IC90, maximum serum dilution that neutralizes 90% of virus.

We next sought to evaluate the immunogenicity of HuAd/RhAd and RhAd/RhAd heterologous prime-boost vaccine regimens. Groups of C57BL/6 mice (*n* = 40 to 50) were primed with 10^9^ vp Ad26-Gag or RhAd52-Gag at week 0. At week 8, mice were boosted (*n* = 8 to 10/group) with 10^9^ vp of ChAd24-Gag, RhAd52-Gag, RhAd53-Gag, RhAd56-Gag, or Ad26-Gag, and CD8^+^ T cell responses were assessed by D^b^/AL11 tetramer binding assays. As shown in Fig. 2A, mice primed with Ad26-Gag were not boosted efficiently by Ad26-Gag due to the antivector preexisting immunity generated by the priming immunization. In contrast, mice primed with Ad26-Gag were robustly boosted with ChAd24-Gag, RhAd52-Gag, RhAd53-Gag, and RhAd56-Gag. In Ad26-Gag-primed mice, the RhAd53-Gag boost induced the highest peak responses of 31.1% at week 9, followed by RhAd52-Gag and RhAd56-Gag. These data show the potency of HuAd/RhAd vaccine regimens. In RhAd52-Gag-primed mice, the RhAd53-Gag and ChAd24-Gag vectors induced the highest responses postboost of 30.5% and 28.4%, respectively, at week 9 (RhAd53-Gag versus Ad26-Gag, *P* = 0.0142; ChAd24-Gag versus Ad26-Gag, *P* = 0.0625), followed by RhAd56-Gag, RhAd52-Gag, and Ad26-Gag. These data show the potency of RhAd/ChAd and RhAd/RhAd vaccine regimens.

We assessed NAb titers to Ad26, RhAd52, RhAd53, and RhAd56 in this experiment following both immunizations. At 4 weeks postprime, only Ad26-Gag-primed mice had Ad26 NAb titers, as expected (Fig. 2B). In contrast, mice primed with RhAd52-Gag had detectable NAb titers against both RhAd52 and RhAd56, indicating a degree of cross-reactive humoral immunity between RhAd52 and RhAd56 (Fig. 2B). Similarly, at 4 weeks postboost, we observed that only mice receiving an Ad26-Gag priming or boosting induced Ad26-specific NAb titers (Fig. 2C). We observed cross-reactive NAb titers among mice boosted with RhAd52-Gag, RhAd53-Gag, and RhAd56-Gag. RhAd52/RhAd52-vaccinated mice generated detectable NAb titers to RhAd53, although RhAd52/RhAd53 generated higher RhAd53-specific NAbs. These data suggest similar potency of these HuAd/RhAd and RhAd/RhAd prime-boost vaccine regimens, despite a degree of cross-reactivity among RhAds.

### Humoral immunogenicity of RhAd vectors in prime-boost regimens.

**FIG 3:**
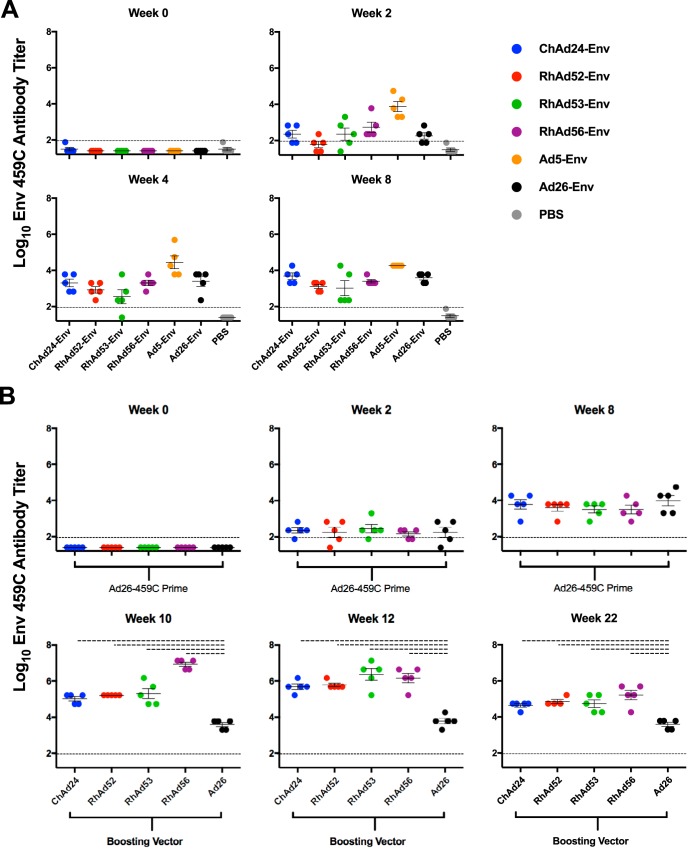
RhAd vector-induced antibody binding titers. (A) C57BL/6 mice were immunized with 10^9^ vp of the indicated adenoviral vectors (*n* = 5/group). Antibody binding titers are shown for weeks 0, 2, 4, and 8 after vaccination. (B) C57BL/7 mice were primed with Ad26-Env and 8 weeks later were boosted with the indicated adenoviral vectors (*n* = 5/group). Antibody binding titers are shown for weeks 0, 2, and 8 postprime and weeks 10, 12, and 22 postboost. Dots represent individual animals. Dotted lines above the graphs denote significance (*P* < 0.01). Error bars indicate the standard error of the mean (SEM).

To investigate the ability of RhAds to induce humoral immunity to the proteins encoded by transgenes, C57BL/6 mice (*n* = 5/group) were immunized with ChAd24-Env, RhAd52-Env, RhAd53-Env, RhAd56-Env, Ad5-Env, or Ad26-Env encoding human immunodeficiency virus type 1 (HIV-1) clade C Env 459C gp140 (28). As shown in Fig. 3A, after a single injection, all vectors induced Env-specific binding antibodies, although Ad5-Env induced faster kinetics and higher peak titers than the other vectors (Fig. 3A). To evaluate prime-boost regimens, C57BL/6 mice (*n* = 5/group) were primed with Ad26-Env at week 0 and boosted with ChAd24-Env, RhAd52-Env, RhAd53-Env, RhAd56-Env, or Ad26-Env at week 8. As shown in Fig. 3B, all groups had similar levels of Env-binding antibody titers postprime, as expected. The Ad26-Env boost did not increase the titers efficiently, presumably as a result of the antivector immunity induced by the priming immunization. In contrast, all of the RhAds efficiently boosted antibody titers. In particular, the Ad26-Env/RhAd56-Env regimen elicited the highest peak antibody titers of 6.93 mean log_10_ titer at week 10 (Ad26-Env versus all other vectors, *P* = 0.0079) (Fig. 3B). These data show that RhAd vectors induce antibody responses, both alone and in the context of prime-boost regimens.

### Effects of HuAd5 preexisting immunity on RhAd vectors.

The extent of immunologic cross-reactivity between the Ad5 and RhAd vectors is not yet known, but it has been previously reported that preexisting Ad5 immunity can impede the immune responses generated by certain nonhuman adenovirus vectors (12). Moreover, as baseline Ad5 seroprevalence is nearly universal in the developing world (1, 6), it is important to evaluate whether high levels of Ad5 preexisting immunity may impact RhAd vector immunogenicity.

**FIG 4:**
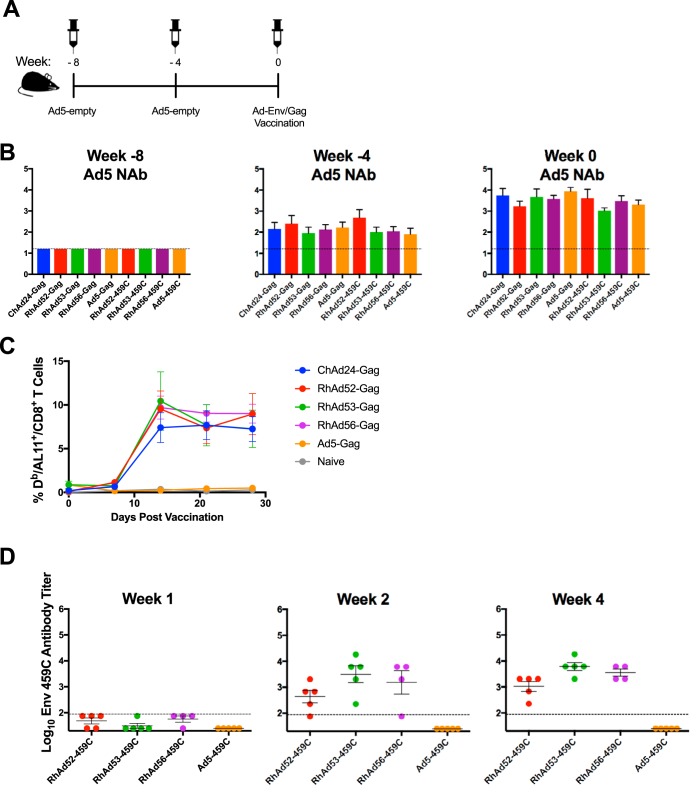
RhAd vector immunogenicity in mice with baseline Ad5 immunity. (A) Experimental schema. C57BL/6 mice (*n* = 50) were immunized at week −8 and week −4 with 10^9^ vp of Ad5-empty. At week 0, mice were injected with the indicated vector expressing either Gag or Env (*n* = 5/group). (B) Ad5-neutralizing antibody titers at weeks −8, −4, and 0. (C) Longitudinal analysis of D^b^/AL11 tetramer binding responses following the priming immunization with the indicated Gag-encoding vector. (D) Antibody binding titers for weeks 1, 2, and 4 after priming immunization with the indicated Env-encoding vectors. Error bars indicate the standard error of the mean (SEM).

To model the effects of Ad5 preexisting immunity on RhAd vector immunogenicity, C57BL/6 mice (*n* = 5/group) were injected twice with 10^9^ vp of Ad5-empty at weeks −8 and −4 (Fig. 4A). As shown in Fig. 4B, these injections raised the median log_10_ Ad5 NAb titers of 3.3 by week 0. At week 0, mice were primed with Ad vectors encoding either simian immunodeficiency virus (SIV) Gag or Env 459C gp140, and the responses were evaluated by D^b^/AL11 tetramer binding assays and Env-specific enzyme-linked immunosorbent assays (ELISAs). As shown in Fig. 4C and D, all RhAds and ChAd24 were unaffected by the presence of high levels of Ad5 preexisting immunity. In contrast, the immunogenicity of Ad5-Gag and Ad5-Env was ablated by high baseline Ad5 NAb titers, as expected.

**FIG 5:**
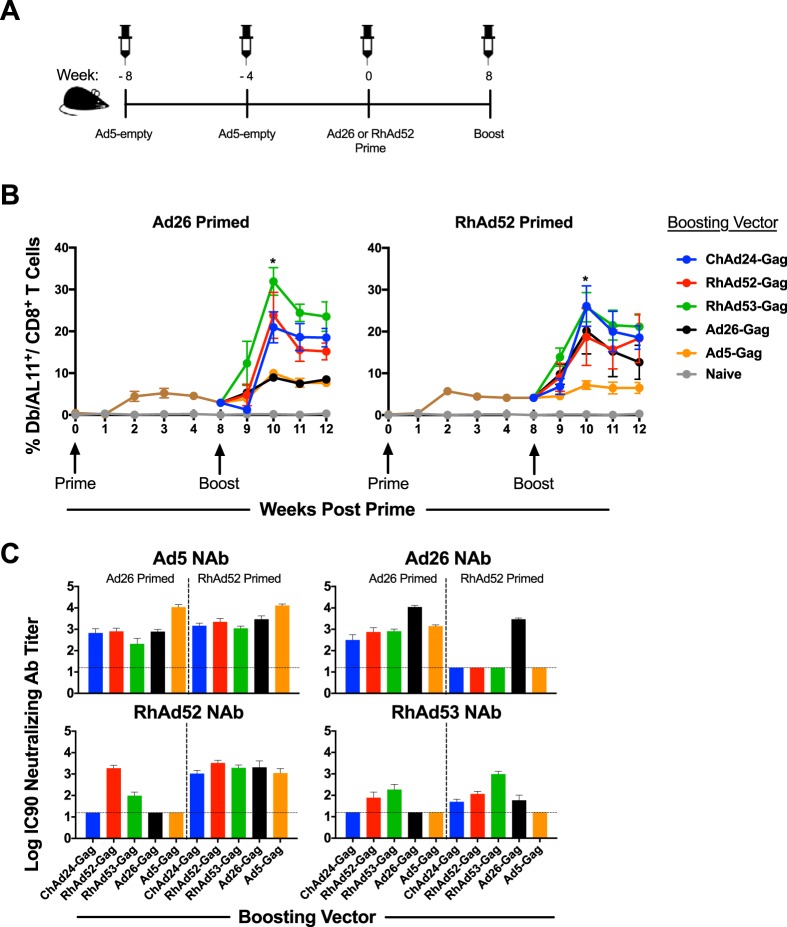
RhAd prime-boost regimens in mice with baseline Ad5 immunity. (A) Experimental study design (*n* = 4/group). C57BL/6 mice were primed with the indicated vector and then boosted 8 weeks later with the boosting vector denoted in the key. Both immunizations were done at 10^9^ vp. (B) Frequency of D^b^/AL11^+^ CD8^+^ T cells. Priming responses were pooled and displayed as one line (brown) for Ad26-Gag and RhAd52-Gag. (C) Ad-specific neutralizing antibody titers 4 weeks after boosting immunization for Ad5, Ad26, RhAd52, and RhAd53. Dotted horizontal lines indicate the limit of detection. Error bars indicate the standard error of the mean (SEM).

We next conducted prime-boost immunization experiments in mice with high levels of baseline Ad5 immunity. C57BL/6 mice (*n* = 40) were preimmunized with two injections of 10^9^ vp Ad5-empty at week −8 and week −4 prior to vaccination (Fig. 5A). All mice had high levels of Ad5 NAb titers following the second Ad5-empty injection (median log_10_ titer, 2.7). At 4 weeks after the second Ad5-empty injection, mice were primed with Ad26-Gag or RhAd52-Gag at week 0 and were boosted with ChAd24-Gag, RhAd52-Gag, RhAd53-Gag, Ad26-Gag, or Ad5-Gag at week 8 (*n* = 4/group). Priming responses were pooled for Ad26-Gag and RhAd52-Gag and depicted on the graph in Fig. 5B as the brown line. As shown in Fig. 5B, Ad5-Gag boosting was poorly immunogenic, presumably due to baseline antivector immunity, and Ad26-Gag boosting was poorly immunogenic, presumably due to the antivector immunity generated by the priming immunization. In contrast, we observed robust boosting by RhAd53-Gag, RhAd52-Gag, and ChAd24-Gag with D^b^/AL11 tetramer binding responses, reaching 31.9% of CD8^+^ T cells at week 10 (RhAd53-Gag versus Ad5-Gag and Ad26-Gag, *P* = 0.028). In RhAd52-Gag-primed mice, all vectors except for Ad5-Gag resulted in effective boosting responses, reaching 26.0% CD8^+^ T cells at week 10; this was particularly the case for the heterologous vectors RhAd53-Gag and ChAd24-Gag (RhAd53-Gag and ChAd24-Gag versus Ad5-Gag, *P* = 0.028). As shown in Fig. 5C, Ad26-specific NAbs were elicited only in mice that received Ad26-Gag either as the prime or boost, and we observed cross-reactivity among the three RhAds. Moreover, as expected, all mice had high levels of Ad5 NAbs due to the Ad5-empty preimmunization. These data demonstrate that high levels of Ad5 preexisting immunity did not impair Ad26/RhAd or RhAd/RhAd prime-boost regimens.

### Impact of cross-reactivity among RhAds on vaccination.

**FIG 6:**
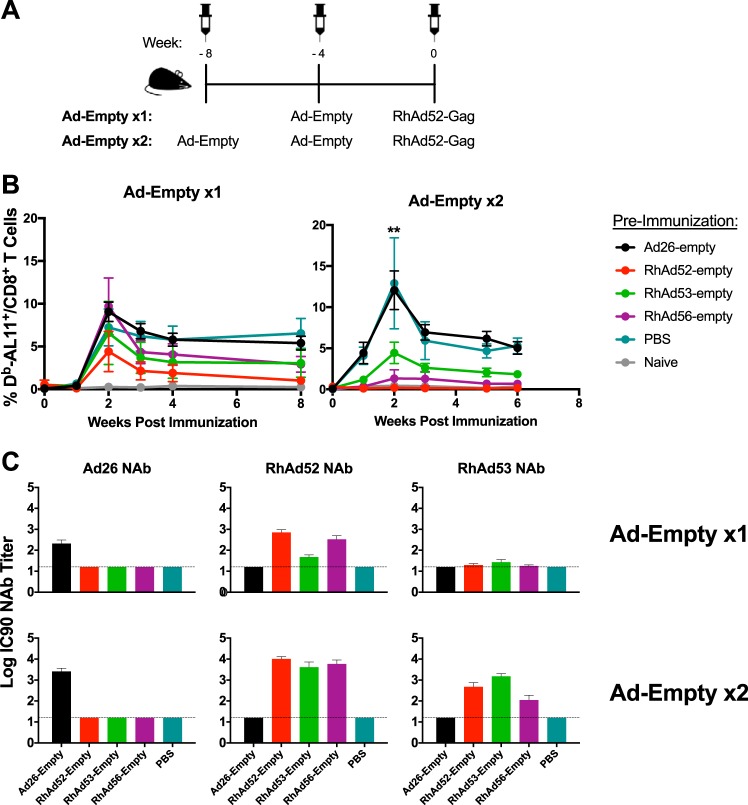
Suppression of RhAd52 immunogenicity with baseline RhAd immunity. (A) Experimental study design (*n* = 5/group). C57BL/6 mice were injected with 10^9^ vp of various Ad-empty vectors either once or twice to induce low or high levels of preexisting immunity. Mice were then vaccinated with 10^9^ vp of RhAd52-Gag. (B) Frequency of D^b^/AL11^+^ CD8^+^ T cells following RhAd52-Gag vaccination following one injection of Ad-empty (left) or two injections of Ad-empty (right). For two injections, *P* was equal to 0.0079 for PBS versus RhAd52 (**), *P* was equal to 0.015 for PBS versus RhAd56 (*), and *P* was equal to 0.055 for PBS versus RhAd53. (C) Neutralizing antibody titers 4 weeks after the first (top) or 4 weeks after the second (bottom) empty vector injection, but before RhAd52 vaccination. Dotted horizontal lines indicate the limit of detection. Error bars indicate the standard error of the mean (SEM).

We next explored the biological significance of the cross-reactive NAb responses among RhAds in this model. C57BL/6 mice (*n* = 5/group) were preimmunized with either one or two injections of 10^9^ vp Ad26-empty, RhAd52-empty, RhAd53-empty, RhAd56-empty, or phosphate-buffered saline (PBS) (Fig. 6A). At 4 weeks after the second Ad-Empty preimmunization, mice received 10^9^ vp of RhAd52-Gag. As seen in Fig. 6B, preimmunization with PBS and Ad26-empty did not blunt the immunogenicity of RhAd52-Gag (PBS versus RhAd52-Gag, *P* = 0.0079; Ad26-Gag versus RhAd52-Gag, *P* = 0.0079). However, we observed substantial suppression of the homologous RhAd52-Gag vector with one RhAd52-empty preimmunization and complete suppression with two RhAd52-empty preimmunizations (Fig. 6B), which raised potent baseline RhAd52 NAbs (Fig. 6C). We also observed minimal attenuation of RhAd52-Gag responses following one RhAd53-empty or RhAd56-empty preimmunization but substantial suppression of RhAd52-Gag following two RhAd53-empty or RhAd56-empty preimmunizations, demonstrating that the cross-reactive NAbs among RhAd vectors can be functionally suppressive if induced to particularly high levels (Fig. 6B and C). Taken together, these data suggest that cross-reactivity among RhAds can suppress a heterologous RhAd vector vaccination when induced to supraphysiologic levels.

### Adoptive transfer studies with purified IgG.

**FIG 7:**
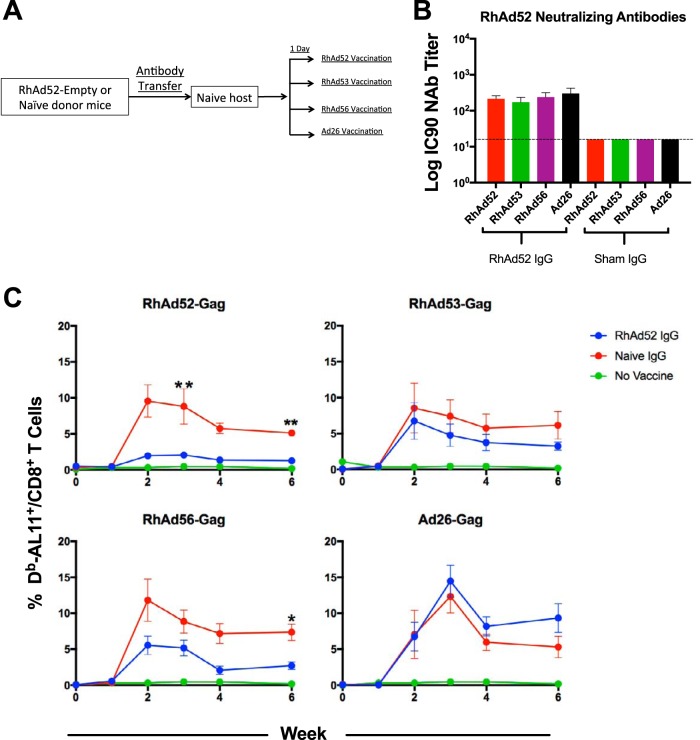
Suppression of RhAd immunogenicity by adoptive transfer of RhAd52-specific IgG. (A) Experimental schema (*n* = 5/group). IgG was purified from pooled serum from mice injected with RhAd52-empty or naive mice, and 500 μg of IgG was transferred to naive recipient mice. One day after transfer, recipient mice were vaccinated with 10^9^ vp of RhAd52-Gag, RhAd53-Gag, RhAd56-Gag, or Ad26-Gag (*n* = 5/group). (B) Neutralizing antibody titers 1 day after adoptive transfer, but before vaccination. (C) Frequency of D^b^/AL11^+^ CD8^+^ T cells following adoptive transfer for each vaccine group (*, *P* < 0.05; **, *P* < 0.01). Error bars indicate the standard error of the mean (SEM).

To explore the suppressive potential of cross-reactive RhAd-specific NAbs in greater detail, we conducted adoptive transfer studies with purified IgG. Donor mice were immunized twice, 4 weeks apart, with 10^9^ vp of RhAd52-empty (Fig. 7A). IgG was then purified from serum and pooled, and 500 μg purified IgG was adoptively transferred into naive recipient mice. As a control, additional groups of recipient mice received IgG purified from unvaccinated control mice. One day after transfer, mice were vaccinated with 10^9^ vp of RhAd52-Gag, RhAd53-Gag, RhAd56-Gag, or Ad26-Gag (*n* = 5/group). Serum collected 1 day after IgG transfer but prior to Ad-Gag vaccination verified RhAd52 NAbs in mice that received RhAd52 IgG but not in mice that received sham IgG (Fig. 7B). As shown in Fig. 7C, RhAd52 IgG nearly completely suppressed RhAd52-Gag (week 2, *P* = 0.0079; week 6, *P* = 0.0079) and partially suppressed RhAd56-Gag (week 6, *P* = 0.0159) but did not significantly impair RhAd53-Gag or Ad26-Gag, thus confirming the suppressive potential of these cross-reactive NAbs.

### Adoptive transfer studies with splenocytes.

**FIG 8:**
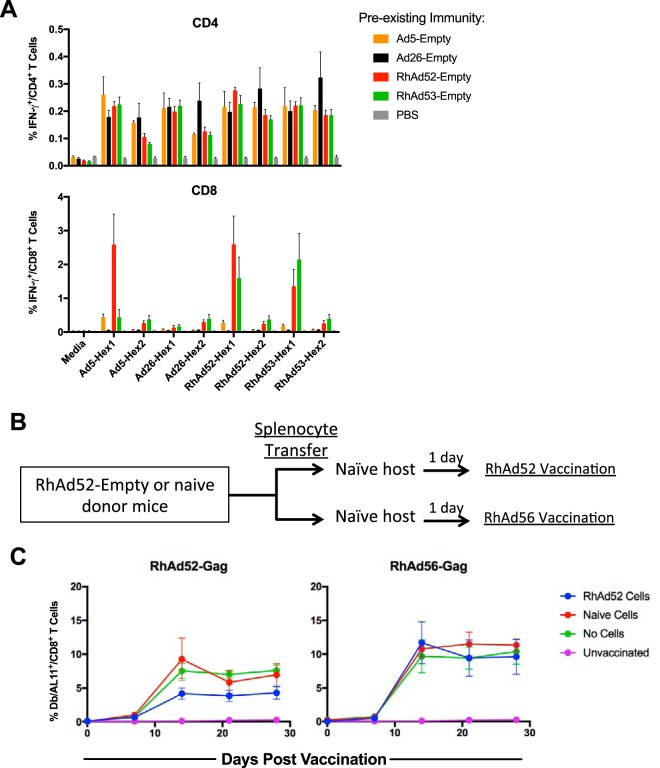
Partial suppression of RhAd immunogenicity by adoptive transfer of RhAd52-specific splenocytes. (A) Frequency of IFN-γ^+^ CD4^+^ and CD8^+^ T cells responding to peptide pools of 15-mers overlapping by 11 amino acids from the hexon regions of Ad5, Ad26, RhAd52, and RhAd53 from mice injected twice with the indicated Ad-empty vector or PBS control. (B) Experimental schema (*n* = 5/group). Splenocytes were pooled from mice that were injected twice with RhAd52-empty or from naive mice. Donor splenocytes were transferred into naive hosts, and 1 day later they were vaccinated with either RhAd52-Gag or RhAd56-Gag (*n* = 5/group). (C) Frequency of D^b^/AL11^+^ CD8^+^ T cells following cell transfer and vaccination. Error bars indicate the standard error of the mean (SEM).

Cross-reactive cellular responses have previously been reported to be extensive among HuAd serotypes (7, 8). To investigate cellular immune cross-reactivity among RhAd vectors, groups of naive C57BL/6 mice (*n* = 5/group) were injected twice with 10^9^ vp of Ad5-empty, Ad26-empty, RhAd52-empty, or RhAd53-empty 4 weeks apart. At 4 weeks after the final injection, spleens were harvested and stimulated with overlapping 15-mer hexon peptides spanning the entire hexon region of each serotype. As shown in Fig. 8A, CD4^+^ T cells exhibited extensive cross-reactivity to homologous and heterologous peptide pools. In contrast, CD8^+^ T cells were more restricted in their cross-reactivity. These data suggest broad cross-reactivity for the CD4 responses and less extensive cross-reactivity for the CD8 responses induced by RhAd vectors.

Finally, we performed a cellular adoptive transfer study to evaluate the biological significance of these cross-reactive T cell responses in this model. C57BL/6 mice were injected twice with 10^9^ vp RhAd52-empty or saline 4 weeks apart, raising median NAb log_10_ titers of 2.3 among the RhAd52-empty groups (Fig. 8B). Groups of naive recipient mice (*n* = 5/group) received 5 × 10^7^ pooled splenocytes from RhAd52-immune or naive donor mice and then were vaccinated with either RhAd52-Gag or RhAd56-Gag. As shown in Fig. 8C, mice that received splenocytes from mice injected with RhAd52-Gag demonstrated a trend toward partial attenuation of the homologous RhAd52-Gag vaccine, but not the heterologous RhAd56-Gag vaccine. These data suggest a modest effect of RhAd-specific cellular immune responses, but the effect is less striking than that of RhAd-specific NAb responses.

## DISCUSSION

In this study, we evaluated the immunogenicity and cross-reactivity of a panel of RhAd vectors, which all cluster phylogenetically into the poorly studied species G of the Adenoviridae (23). We demonstrated that these RhAd vectors are highly immunogenic in the presence of high levels of preexisting HuAd-specific immunity and can be combined into potent HuAd/RhAd and RhAd/RhAd prime-boost vaccine regimens. Moreover, we defined a degree of cross-reactive NAbs among the RhAds as well as extensive cellular cross-reactivity between HuAds and RhAds. Nevertheless, the RhAd/RhAd prime-boost regimens remained highly immunogenic, although suppression by supraphysiologic titers of cross-reactive NAbs could impair the immunogenicity. These data demonstrate the immunogenicity of RhAd vectors and their potential utility as candidate vaccine vectors.

Species G of Adenoviridae remains poorly characterized compared to the other species of adenoviruses (18). Since its classification, only one human adenovirus, HuAd52, has been assigned to this species, and interestingly, HuAd52 was isolated from a primary monkey cell line (29). All the RhAds discovered to date have clustered into species G (18, 23). Our data show that the RhAds induce a cellular immune phenotype more similar to that induced by Ad26 than by Ad5 and are highly immunogenic, despite high levels of HuAd preexisting immunity (Fig. 1, 2, and 5). To our knowledge, this is the most in-depth assessment of the vaccine-elicited immune responses by species G-based adenoviral vectors.

Preexisting immunity to adenoviral vectors, particularly baseline NAbs, has been shown to suppress the immunogenicity of Ad vectors (1, 4, 5, 16). These NAbs are typically serotype specific, although we previously identified cross-reactive NAbs between human Ad11 and Ad35 (16). Here we observed a degree of cross-reactive NAbs between RhAd52, RhAd53, and RhAd56, although the titers of the cross-reactive NAbs were lower than those of the homologous NAbs, and the cross-reactive NAbs did not impair the immunogenicity of RhAd/RhAd prime-boost regimens (Fig. 2 and 5) unless they were induced to very high titers (Fig. 6). The extent of cross-reactivity among the RhAds reflected their phylogenetic relatedness (Fig. 1A). For example, RhAd52 and RhAd56 are more closely related in terms of their full genomes and more readily induce cross-reactive NAbs to each other than to RhAd53. RhAd seroprevalence is exceedingly low in the human population, and NAb titers, when present, are very low (18).

Cellular immune responses to adenoviruses are extensively cross-reactive across serotypes (7, 14, 15) and may have a secondary role in suppressing vaccine-elicited immune responses (3, 7, 16). We show here that RhAds conform to this paradigm as well, with broad CD4 T cell cellular cross-reactivity and more limited CD8 T cell cross-reactivity. However, cross-reactive NAbs are likely more critical than cross-reactive cellular responses in attenuating vector immunogenicity (Fig. 7 and 8) (16).

In conclusion, our data demonstrate the potent immunogenicity of RhAd vectors in mice. We observed a limited degree of humoral cross-reactivity and extensive cellular cross-reactivity among RhAd vectors. Nevertheless, HuAd/RhAd and RhAd/RhAd prime-boost regimens were highly immunogenic, and all the RhAds effectively circumvented high levels of baseline Ad5-specific immunity. In addition, the use of these RhAd vectors expands the ability to vaccinate against multiple pathogens in a population that may have already received HuAd or ChAd vectors. These data suggest the potential of RhAd vectors and prime-boost regimens as candidate vaccines.

## MATERIALS AND METHODS

### Phylogenetic trees.

Phylogenetic trees were constructed using MEGA (version 7) software (www.megasoftware.net). Whole-genome and hexon DNA sequences were aligned using the ClustalW program. Maximum likelihood phylogenetic trees were based on the general time-reversible model and were bootstrapped 50 times. The trees with the highest log likelihoods are shown. The tree is drawn to scale, with branch lengths measured in the number of substitutions per site.

### Mice and immunizations.

Female C57BL/6 mice (The Jackson Laboratory) were used for all immunization experiments. Mice were vaccinated with the E1/E3-deleted Ad5, Ad26, ChAd24, RhAd52, RhAd53, or RhAd56 vector (18). The vectors either were empty (containing no transgene) or expressed SIV_mac_239 Gag or HIV-1 clade C Env 459C gp140 (28) transgenes and were injected intramuscularly in the quadriceps at a dose of 10^9^ viral particles in a volume of 100 μl divided equally between the two legs. All animal experiments were performed in accordance with Beth Israel Deaconess Medical Center Institutional Animal Care and Use Committee guidelines.

### ELISA.

Enzyme-linked immunosorbent assays (ELISAs) were performed as described previously (30). Briefly, ELISA plates (Thermo Scientific) were coated overnight at 4°C with HIV-1 clade C Env 459C gp140. On the following day, mouse serum was added to the plates and serially diluted. After a 1-h incubation, horseradish peroxidase (HRP)-conjugated rabbit anti-mouse immunoglobulin secondary antibody (Jackson ImmunoResearch Laboratories) was added to the plates for another 1-h incubation. Finally, the plates were developed and analyzed using a SpectraMax Plus ELISA plate reader (Molecular Devices) and Softmax Pro-6.5.1 software. Endpoint titers were determined to be positive at the highest dilution that maintained an absorbance greater than 2-fold above the background levels.

### Mouse tissue processing and flow cytometry.

Mice were bled submandibularly, and PBMCs from whole blood were isolated using Ficoll-Hypaque density centrifugation at 1,900 rpm for 20 min. Spleens were processed as previously described (24). Major histocompatibility complex class I tetramer staining was performed using the H-2D^b^ tetramer loaded with the immunodominant AL11 peptide (AAVKNWMTQTL) as described previously (24). Biotinylated class I monomer was provided by the National Institutes of Health Tetramer Core Facility (Emory University, GA). PBMCs were surfaced stained with anti-PD-1 (clone RMP1-30), anti-CD8a (clone 53-6.7), anti-CD44 (clone IM7), and anti-KLRG1 (clone 2F1).

Splenocytes were stimulated with 1 μg/ml of an overlapping SIV_mac_239 Gag peptide pool. At the time of stimulation, brefeldin A (BD Biosciences) was added and samples were incubated for 5 h at 37°C. After the incubation, cells were washed and stained with the surface stain antibodies (mentioned above), permeabilized with Cytofix/Cytoperm (BD Biosciences), and stained with anti-IFN-γ (clone XMG1.2) antibodies for half an hour. Vital exclusion dye was purchased from Invitrogen. All antibodies were purchased from either BioLegend or BD Biosciences. All samples were acquired using an LSR II flow cytometer (BD Biosciences), and data were analyzed using FlowJo (version 9.6.4) software (TreeStar).

### Neutralization assays.

Adenovirus-specific neutralization antibody (NAb) titers using mouse serum samples were conducted as previously described (31). Briefly, serum was 2-fold serially diluted in a 96-well flat-bottom plate, with the exception of the last column, which served as the maximum-infection control. Replication-incompetent recombinant Ad-Luc reporter construct viruses were added to the plate, followed by the addition of A459 cells. The plates were incubated for 24 h at 37°C in 10% CO_2_. After incubation, the medium was removed and 100 μl of phosphate-buffered saline (PBS) and 100 μl of Steady-Glo substrate (Promega) were added to the wells. The luciferase (Luc) activity in the cells was measured with a Victor 3 multilabel counter (PerkinElmer, Waltham, MA). Neutralization titers were defined as the maximum serial dilution where 90% of the virus was neutralized by the serum.

### IgG purification.

IgG was purified from mouse serum using IgG purification NAb spin kits (Thermo Scientific) according to the manufacturer's instructions. Serum was bound to the spin column and washed with binding buffer (Thermo Scientific). Bound IgG was then eluted using elution buffer (0.1 M glycine, pH 2 to 3) and neutralized with neutralization buffer (1 M Tris, pH 8.5 to 9). IgG was then buffer exchanged into 1× PBS via spin columns (Amicon Ultra 10k device).

### Adoptive transfers.

Adoptive transfer studies were performed essentially as previously described (13). Donor mice were immunized twice, 4 weeks apart, with RhAd52-empty to generate baseline vector immunity. Recipient mice received either 5 × 10^7^ splenocytes or purified IgG from either the RhAd52 donor mice or naive donor mice via the intravenous route. One day following adoptive transfer, mice were vaccinated with the RhAd52, RhAd53, RhAd56, or Ad26 vector expressing SIV Gag. Following vaccination, mice were followed weekly for tetramer binding responses as mentioned above.

### Statistical analysis.

Statistical analyses were performed using a two-tailed nonparametric Mann-Whitney U *t* test and GraphPad Prism (version 7.0) software (GraphPad Software).
